# Differential strengths of selection on S-RNases from *Physalis *and *Solanum *(Solanaceae)

**DOI:** 10.1186/1471-2148-11-243

**Published:** 2011-08-19

**Authors:** Timothy Paape, Joshua R Kohn

**Affiliations:** 1 Department of Plant Biology, University of Minnesota, 250 Biological Science Center, 1445 Gortner Ave. St. Paul, MN 55108, USA; 2 Section of Ecology, Behavior and Evolution, Department of Biological Sciences, University of California San Diego, 9500 Gilman Drive, La Jolla CA 92093-0116, USA

**Keywords:** positive selection, non-synonymous and synonymous substitution, S-RNase, polymorphism, *Physalis, Solanum*

## Abstract

**Background:**

The S-RNases of the Solanaceae are highly polymorphic self-incompatibility (S-) alleles subject to strong balancing selection. Relatively recent diversification of S-alleles has occurred in the genus *Physalis *following a historical restriction of S-allele diversity. In contrast, the genus *Solanum *did not undergo a restriction of S-locus diversity and its S-alleles are generally much older. Because recovery from reduced S-locus diversity should involve increased selection, we employ a statistical framework to ask whether S-locus selection intensities are higher in *Physalis *than *Solanum*. Because different S-RNase lineages diversify in *Physalis *and *Solanum*, we also ask whether different sites are under selection in different lineages.

**Results:**

Maximum-likelihood and Bayesian coalescent methods found higher intensities of selection and more sites under significant positive selection in the 48 *Physalis *S-RNase alleles than the 49 from *Solanum*. Highest posterior densities of dN/dS (ω) estimates show that the strength of selection is greater for *Physalis *at 36 codons. A nested maximum likelihood method was more conservative, but still found 16 sites with greater selection in *Physalis*. Neither method found any codons under significantly greater selection in *Solanum*. A random effects likelihood method that examines data from both taxa jointly confirmed higher selection intensities in *Physalis*, but did not find different proportions of sites under selection in the two datasets. The greatest differences in strengths of selection were found in the most variable regions of the S-RNases, as expected if these regions encode self-recognition specificities. Clade-specific likelihood models indicated some codons were under greater selection in background *Solanum *lineages than in specific lineages of *Physalis *implying that selection on sites may differ among lineages.

**Conclusions:**

Likelihood and Bayesian methods provide a statistical approach to testing differential selection across populations or species. These tests appear robust to the levels of polymorphism found in diverse S-allele collections subject to strong balancing selection. As predicted, the intensity of selection at the S-locus was higher in the taxon with more recent S-locus diversification. This is the first confirmation by statistical test of differing selection intensities among self-incompatibility alleles from different populations or species.

## Background

Self-incompatibility (SI) polymorphisms are maintained by balancing selection over long evolutionary time scales. Selection continually favors rare alleles because they are less frequently rejected as mates [1,2]. Shared ancestral polymorphism is commonly observed as a result of strong balancing selection with alleles from different species and genera clustering together in phylogenetic reconstructions [3-5]. This implies that S-alleles are often much older than the species from which they are sampled. Coalescence times of S-locus polymorphisms are often estimated as a few tens of millions of years, far longer than coalescence times of polymorphism at loci not subject to balancing selection [6,7]. Sequence divergence at S-loci is also extreme, with stylar S-alleles often differeing at 40% or more of their amino acids. This is another sign of their great age, as well as the rarity of recombination at known S-loci. Also of importance for the current study, alleles undergoing diversification can leave distinct signatures of positive selection among amino acid sites across related taxa.

Richman et al. [3] detected a remarkable reduction in the extent of shared ancestral polymorphism among alleles from the S-RNase locus, which encodes the stylar specificity component of the gametophytic SI system of Solanaceae. In particular, *Physalis crassifolia *alleles, while numerous, all belonged to just three trans-generic lineages while alleles sampled from most other Solanaceae represented far more ancient lineages. Estimates of historical effective population sizes of *Solanum carolinense *and *P. crassifolia *showed at least an order of magnitude decrease in *Physalis *relative to *Solanum *[3]. The pattern found in *P. crassifolia*, in which all S-alleles within the species represent only three ancient lineages, is shared by other SI *Physalis *species and by SI members of the closely related genus *Witheringia *[7-14]. These findings have been interpreted as the result of a historical restriction of S-locus diversity that occurred approximately 15 MYA [7] in a common ancestor of *Physalis *and *Witheringia *that is not shared with *Solanum *or other sampled genera of Solanceae [3,7,13].

Genealogical patterns suggest that *Physalis *S-RNase alleles underwent rapid re-diversification following the historical restriction at the S-locus [8,13,14]. Because allele numbers in *Physalis *species are comparable to those found among species of *Solanum*, it is thought that post-bottleneck rediversification has returned allele numbers to equilibrium or nearly so [3]. This provides an opportunity to examine patterns of selection on sets of S-RNase alleles that have different evolutionary histories. The more recently diversified S-alleles of *Physalis *might be expected to show greater rates of non-synonymous substitutions because of the increased strength of recent diversifying selection [2]. The intensity of selection on S-alleles is inversely proportional to their number. So when the number of alleles is below equilibrium, as after a severe bottleneck, selection intensity is predicted to be higher than it is after equilibrium in allele number is achieved [2]. The time frame over which a period of heightened selection would be evident at the self-incompatibility locus is not known.

Here we compare selective regimes acting on the S-RNase alleles drawn from species of *Physalis *and *Solanum *(Solanaceae). Positive selection has been estimated among self-incompatibility alleles of several taxa using various methods [13,15-19], most commonly the maximum likelihood phylogenetic approaches first proposed by Nielsen and Yang [20] and more recently by coalescent-based methods described by Wilson and McVean [21]. These methods use the ratio of non-synonymous (dN) to synonymous (dS) nucleotide substitutions (ω) to estimate patterns of selection at individual codons. In this study, we investigate positive selection on amino acids among S-RNases both within and across species of *Physalis *and *Solanum *(Solanaceae). These polymorphic S-alleles provide useful contrasts because diversification at the S-locus in the different genera took place during different time periods and among different S-allele lineages.

Several previous studies [19,22,23] have utilized PAML [20] to assess which codons within S-allele sequences were subject to positive selection in different taxa. However, none of these studies have been able to statistically determine how the strength and location of selection differs between groups of sequences. For instance, Castric and Vekemens [19] compared patterns of selection among several taxa at the S-receptor kinase (*SRK*) locus which controls stylar recognition in the sporophytic SI system found in Brassicaceae. Using PAML on separate datasets from each taxon, a higher intensity of selection (higher ω) was estimated among positively selected sites in *Brassica *relative to those in two self-incompatible species of *Arabidopsis*. This was attributed to post-bottleneck diversification of *SRK *alleles in *Brassica*. However, given the methods used, the statistical significance of the difference in estimates of selection intensity could not be evaluated.

PAML analyses [19] also found different sites under significant positive selection in different sets of S-alleles. It was concluded, however, that this was poor evidence for selection occurring on different sites. In their study [19], the power to detect selection was shown to be low so non-overlap in the codons found to be under selection in different datasets would be expected, even if selection acted on the same sites in each set of alleles. Similarly, Vieira et al. [22] looked at positive selection across S-RNases and found evidence for different positively selected sites in S-RNases from different families and sub-families of flowering plants. Again however, they did not employ a statistical framework capable of testing the significance of differences in selective pressures acting on the same codons in different taxa.

In this study we apply both phylogenetic maximum likelihood and coalescent Bayesian methods, treating S-allele alignments and phylogenies from species in each genus either as a) distinct datasets compared using a series of nested maximum likelihood and Bayesian models of selection or b) as a combined data set in which specific clades of interest within single phylogenies are examined. Our primary goal is to apply statistical frameworks using formal hypothesis tests to answer the following questions: 1) Can we detect significant differences in the strength of selection between genera? 2) Do the proportions of sites under selection differ among genera? 3) Which sites show significantly different selection intensities between genera? 4) Are differences in the strength of selection due to significantly higher dN or dS in one dataset relative to the other? 5) Do sites under selection differ among S-allele lineages?

## Results

**Figure 1:**
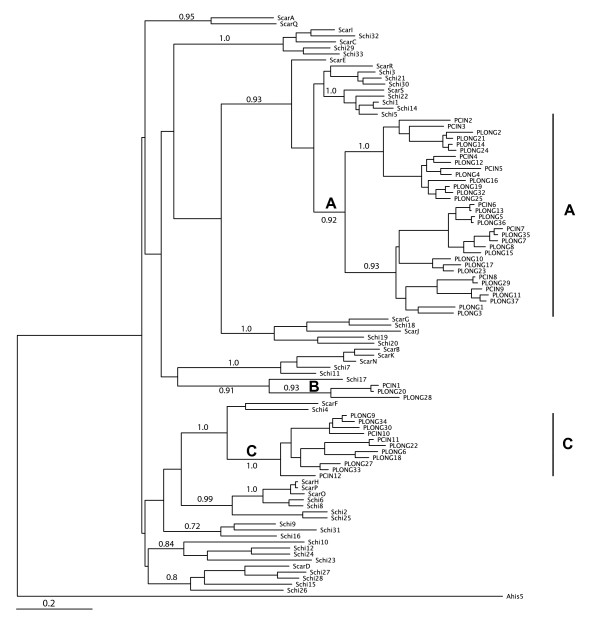
**Phylogeny of *Physalis cinerascens *(Pcin), *P. longifolia *(Plong), *Solanum carolenense *(Scar), and S. *chilense *(Schi) S-RNases**. Posterior probability scores show branch support for lineages of interest. The restricted (bottlenecked) lineages of *Physalis *are indicated at branches A, B and C. The phylogeny was created using Mr. Bayes v3.1 (Ronquist and Huelsenbeck 2003).

A Bayesian consensus phylogeny of S-alleles from *Physalis *and *Solanum *is shown in Figure 1. The three ancient *Physalis *lineages (clades A, B and C in Figure 1) are consistent with previously published topologies [7,11,14] that use S-alleles from more genera and illustrate re-diversification from within only those lineages. No *Solanum *alleles are found within those lineages. Estimates of average pairwise nucleotide diversity (π) show synonymous divergence is greater for *Solanum *while non-synonymous divergence is similar among the genera (Table 1). A greater accumulation of synonymous substitutions is expected for *Solanum *S-alleles if these lineages are older than those of *Physalis *as suggested by previous studies [3,7,13].

**Table 1:** Average pairwise nucleotide divergence (π) among S-alleles for each species and genus estimated using DNASP 4

Taxa (*n *alleles)	**Synonymous (**π**s)**	**Non-Synonymous (**π**n)**	All Sites
*Physalis *(47)	0.33	0.34	0.33
*P. cinerascens *(12)	0.37	0.37	0.37
*P. longifolia *(37)	0.33	0.33	0.33

*Solanum *(49)	0.48	0.34	0.37
*S. carolinense *(17)	0.47	0.35	0.38
*S. chilense *(32)	0.5	0.34	0.37

### Do selection intensities or the proportion of sites under selection differ among S-alleles from *Solanum *versus *Physalis*?

A random effects likelihood (REL) approach [24,25] was used to compare the distributions of non-synonymous (dN) and synonymous substitutions (dS) across genera and found that they differed significantly in three of four likelihood ratio tests (LRTs; Table 2). The alternative hypothesis (H_A_) where dN and dS were free to vary had the highest log-likelihood score (lnL **= -**16749.63). The estimated dN/dS ratio for the positively selected class of codons in *Physalis *alleles under this model was roughly twice that estimated from *Solanum *alleles (*Physalis *dN/dS 2.663, *Solanum *dN/dS 1.139, Table 1). The null model (a) that constrains both datasets to have equivalent dN/dS ratios for the class of sites under positive selection is strongly rejected (*p *< 0.0001; df = 1) while the null model (b) constraining the proportions of selected sites across datasets was not rejected (*p *< 0.165; df = 1). This test allows dN/dS ratios of selected sites from the two genera to vary freely but enforces the proportions (*p*_1 _and *p*_2_) in the positive selection class to be equal. The selective regime test (c), which constrains dN/dS ratios for the positively selected sites and the proportion of selected sites to be equal across both genera, was also strongly rejected (*p *< 0.001; 1df). Rejection of this model is unlikely to be due to variation in proportions of selected sites based on the results of (b) and appears largely the result of differences in the strength of selection on positively selected sites across datasets. The shared distributions test (d) combines the joint distributions of dN and dS for both datasets and was also found to have a significantly lower likelihood (*p *< 0.001; 10 df) than H_A _which allows for variation in rates in both datasets. See Methods for full descriptions of each model. To summarize, the REL approach found significantly greater intensity of selection on positively selected sites in *Physalis *but no evidence that the proportion of sites under selection differed between genera.

**Table 2:** Comparative rate distribution tests of non-synonymous and synonymous substitutions across datasets

H_A_: Rates free to vary							
Log likelihood: -16749.63				Parameters: 229			
Inferred rates for *Physalis: *				Inferred rates for *Solanum:*			
dN/dS	dS	dN	Prob	dN/dS	dS	dN	Prob

2.663	1.047	2.788	0.463	1.139	0.942	1.073	0.353
1.000	0.814	0.814	0.311	1.000	2.000	2.000	0.094
0.000	0.580	0.000	0.081	0.496	0.800	0.397	0.274
0.177	1.487	0.262	0.144	0.083	0.933	0.077	0.279

**a) H_0_: Same strength of selection**							
**Log likelihood: -16765.49**				Parameters: 228			

Inferred rates for *Physalis*:				Inferred rates for *Solanum*:			
dN/dS	dS	dN	Prob	dN/dS	dS	dN	Prob

1.664	1.261	2.099	0.466	1.664	0.781	1.300	0.337
1.000	0.647	0.647	0.312	1.000	2.241	2.241	0.086
0.000	0.470	0.000	0.081	0.527	0.884	0.466	0.290
0.171	1.222	0.209	0.141	0.087	1.001	0.087	0.286

Are selection strengths (dN/dS) different?							
**LRT = 31.722 p < 0.0001**; DF = 1							
**b) H_0_: Same proportion of selected sites**							
**Log likelihood: -16750.60**				Parameters: 228			

Inferred rates for *Physalis*:				Inferred rates for *Solanum*:			
dN/dS	dS	dN	Prob	dN/dS	dS	dN	Prob

2.737	1.042	2.851	0.397	1.143	0.949	1.085	0.397
1.000	0.900	0.900	0.339	1.000	2.065	2.065	0.081
0.000	0.573	0.000	0.084	0.491	0.804	0.395	0.258
0.216	1.297	0.280	0.180	0.082	0.939	0.077	0.264

Are the proportions of codons under selection different?							
LRT = 1.929 p < 0.165; DF = 1							
**c) H_0_: Same dN/dS and proportions**							
**Log likelihood: -16766.96**				Parameters: 228			

Inferred rates for *Physalis*:				Inferred rates for *Solanum*:			
dN/dS	dS	dN	Prob	dN/dS	dS	dN	Prob

1.636	1.318	2.157	0.397	1.636	0.805	1.318	0.397
1.000	0.703	0.703	0.348	1.000	2.341	2.341	0.074
0.000	0.472	0.000	0.087	0.517	0.894	0.463	0.265
0.193	1.136	0.219	0.169	0.086	1.022	0.088	0.264
Are selective regimes (dN/dS and proportions) different?							
**LRT = 34.647 p < 0.0001**; DF = 2							
**d) H_0_: Shared distributions of rates**							
**Log likelihood: -16764.30**				Parameters: 219			
				
Inferred joint rates:							
dN/dS	dS	dN	Prob				
				
2.507	1.034	2.593	0.189				
1.000	1.139	1.139	0.338				
0.543	0.797	0.433	0.251				
0.086	0.988	0.085	0.222				
			
Are the distributions different?							
**LRT = 29.350 p < 0.001**; DF = 10							

NOTE: Null models (a-d) were tested using likelihood ratio tests (LRTs) against the alternative model H_A _where dN and dS rates are free to vary in each dataset. Significance of *p *≤ 0.05 was determined using χ^2 ^with degrees of freedom (DF) equal to the number different parameters between models

### Which sites show significant differences in strengths of positive selection?

**Figure 2:**
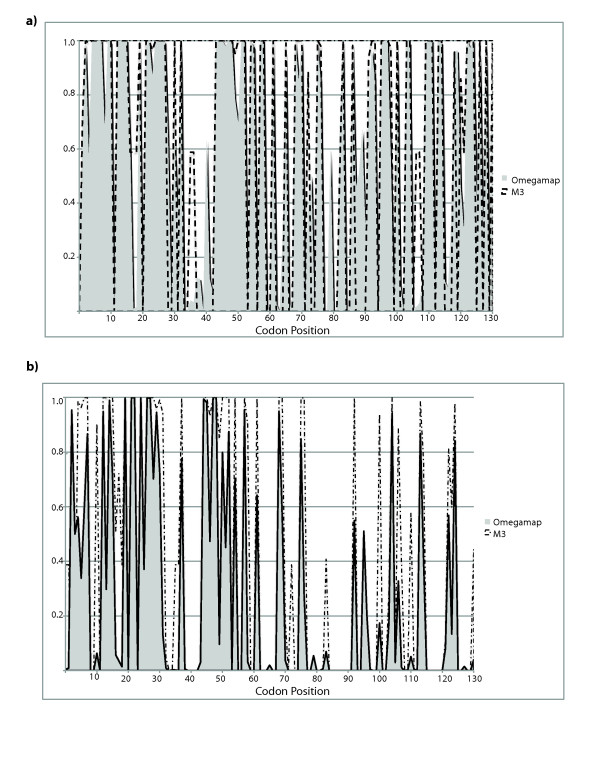
**Posterior probability scores of sites predicted to be under positive selection in a) *Physalis *and b) *Solanum *using OmegaMap (Wilson and McVean 2006) (gray) and the general discrete model M3 (dashed lines) of Nielsen and Yang (1998)**.

Because the REL approach used above does not indicate which codons show different dN/dS ratios, subsequent analyses were conducted to determine where along the S-RNase sequence selection differs between genera. We first estimated positive selection at individual codons using the Nielsen and Yang [20] method implemented in PAML v3.15. These results detected considerably more positively selected codons in *Physalis *than *Solanum *as indicated by posterior probabilities > 0.99 (Figure 2). Because we cannot determine whether the selective regime at these sites differs significantly between datasets under the current framework of the maximum likelihood method implemented in PAML, we employed a Bayesian coalescent method described by Wilson and McVean [21] to compare highest posterior densities (HPDs) for point estimates of ω (= dN/dS). We first compared our results from OmegaMap with the Nielsen and Yang M3 model for both datasets to determine how similar were the estimates of which codons were under positive selection. Posterior probability scores show consistent trends across methods for each dataset (Figure 2), though some sites have higher scores using M3 in *Solanum*. Most importantly, both methods identify nearly all of the same sites under positive selection upon which to estimate ω values. Wilson and McVean [21] suggested that inconsistencies between their coalescent method results for estimating ω and those of *codeml *in PAML could be the result of recombination. We did not detect the presence of recombination in either dataset using the likelihood permutation test described by McVean et al. [26] (results not shown).

**Figure 3:**
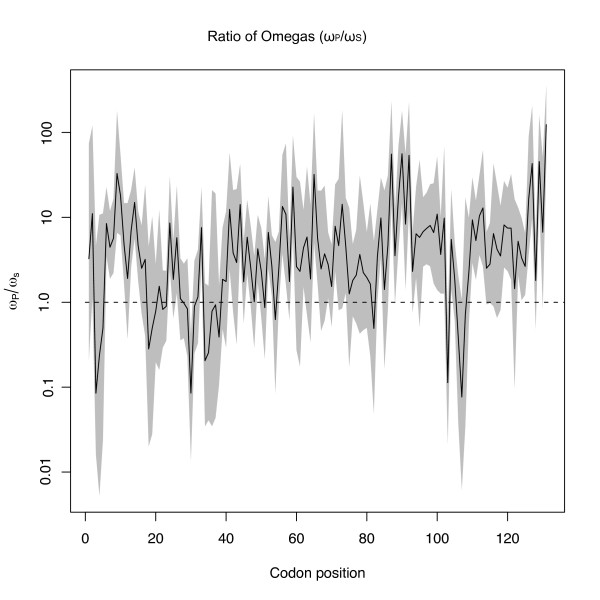
**Bayesian estimate of the ratio of omega values (ω_P _= *Physalis *d_N_/d_S_; ω_S _= *Solanum *d_N _d_S_) for each codon position**. The gray region is the 95% highest posterior density (HPD) and the solid line is the mean of the ratios. If the HPD crosses the value 1 (dashed line) then the ratios are not significantly different. HPD's above the line indicate a higher ω for *Physalis *than *Solanum *S-alleles.

**Figure 4:**
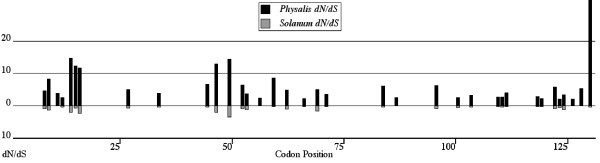
**Contrast of point estimates of dN/dS for *Physalis *and *Solanum *for sites that were found to have omega ratios (ω_P/_ω_S_) significantly above 1 (from Figure 2)**. Sites indicated were first determined to be positively selected in at least one dataset based on posterior probability scores > 0.95 for both PAML and OmegaMap. For all sites, *Physalis *had higher estimated dN/dS ratios.

To compare selection intensities at specific sites across genera, estimates of the mean and upper and lower highest posterior densities (HPD's) for ω from each dataset were used to generate distributions from 500,000 MCMC iterations of the ratio of ω values from *Physalis *and *Solanum *(Figure 3). Confidence intervals (HPD's) that do not include 1 (dotted line in Figure 3) indicate that the codon specific estimates of ω from each dataset (ω_p _and ω_s _for *Physalis *and *Solanum*, respectively) are significantly different. The HPDs of ω_P_/ω_S _ratios are more heavily concentrated in the upper half of Figure 3 (above dashed lined) indicating that codons from *Physalis *generally have higher dN/dS ratios than those from *Solanum*. Significantly different ω values are found at 57 positions. Not all 57 sites with ω_P_/ω_S _ratios significantly > 1showed significant posterior probabilities of being under positive selection (dN/dS > 1) when genera were analyzed separately. We therefore removed sites that, for neither genus, showed ≥ 0.95 posterior probabilities of positive selection using either OmegaMap or PAML (Figure 2). That is, we removed sites showing no strong evidence of being under positive selection in either genus. Of the remaining sites, all but 3 had posterior scores ≥ 0.99 for ω > 1. Thirty-six sites had significantly higher ω_P_/ω_S _ratios and posterior probabilities ≥ 0.99 for *Physalis *(Figure 4). By the same criteria, no sites showed significantly stronger selection in *Solanum *relative to *Physalis*.

**Figure 5:**
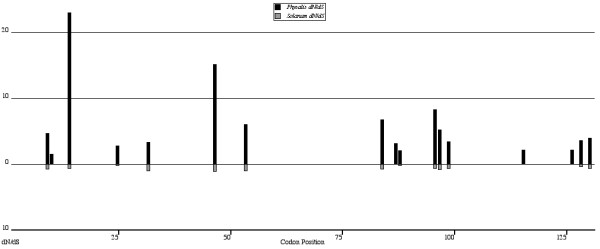
**Fixed effects likelihood (FEL) comparisons of non-synonymous (dN) substitutions at sites predicted to be under significantly different selection pressure (*p ≤ *0.05)**. A total of 17 sites differed, with all determined to be greater for *Physalis *than *Solanum*.

We also used a fixed effects likelihood (FEL) method [27] to compare selective pressures (FEL-CSP) at individual sites across data sets. Like the Bayesian coalescent method, we used independent phylogenies for each genus, then statistically compared individual codons across taxa under a hypothesis testing scheme (see Methods). This method also finds several codons in *Physalis *that are under significantly greater positive selection than *Solanum *as shown by contrasts of mean dN/dS values at these particular sites (Figure 5). FEL-CSP identified fewer differentially selected sites than the Bayesian method with 16 sites predicted to be differentially selected at the *p *≤ 0.05 level and one site with *p *= 0.08. All but six of these sites were also identified by the coalescent method (Table 3). Because this method does not utilize rate distributions across sites, it is sensitive to the number of taxa present in each dataset [28]. We performed a power analysis to determine whether *p*-values ≤ 0.05 were sensitive to potential type II errors for the FEL analysis. We found that that the power to detect positively selected sites for *Physalis *is only 39.4%, and 34% for *Solanum *at *p *= 0.05. However, the false positive rate for sites predicted under this method is also low, 4.3% and 4.9% for *Physalis *and *Solanum *respectively. This means that when a site is predicted to be under selection, accuracy of this prediction is expected to be ≥ 95%.

**Table 3:** Sites predicted to be under differential positive selection using the Bayesian ratio of omegas (ω_1_/ω_2_) test, the fixed effects likelihood comparison of selective pressures (FEL-CSP), and FEL-Clade tests

S-RNase Region (codon positions)						
**Test**	**HVa (1-38)**	**HVb (44-62)**	**C3 (63-68)**	**V1 (69-84)**	**C4 (85-93)**	**V2 (94-131)**

ωp/ωsα *Physalis *codons	7,**8**, 10, 11, **13**,14, 15, 26, 33	44, **46**, 49 52, **53**, 56 (0.97), 59 62	- - - -	69, 71, **84**	**87 **(0.98)	**9**, 101, 104, 110, 111, 112, 119 (0.96), 120 (0.96), 123, 124 (0.97), **125** **127 **(0.95), **129, 131**

FEL CSPb *Physalis *codons	**8**, 9,**13**, 24, 26 (0.08), 31	**46, 53**	-	84	**87**	**96**, 99, 116, **125** **127, 129, 131**

FEL Clade A *Physalis *codons	3, 6, 9, 10, 13	46, 56	-		87, 89, 90	96, 99, 110, 121

FEL Clade C *Physalis *codons	9, 14, 24	44, 57	-		92	97, 127, 129

FEL Clade Sc *Solanum *codons	21, 22, 23, 29 30, 32, 34, 35, 38	60	Clade A as forground -	77		103, 106, 109

FEL Clade Sc *Solanum *codons	23, 31	47, 48, 50	Clade C as forground -	80		124

Bold indicates sites predicted to be under stronger selection in *Physalis *than in *Solanum *by both ω_p_/ω_s _and FEL CSP tests. FEL-Clade tests comparing clades A and C show mostly different sites under selection as well as several different sites selected in *Solanum *depending on the foreground *Physalis *clade present.

a) For the ω_1_/ω_2 _test codons had a posterior probability score ≥ 0.99 unless otherwise indicated in parentheses.

b) For the FEL test all sites listed had a *p-*value ≤ 0.05 except site 26.

c) The FEL Clade Tests had either Clade A or Clade C as the foreground with all other *Physalis *alleles removed from the alignment and phylogeny.

### Do different S-allele lineages experience greater selection intensities?

To test whether a branch or clade model fits the data better than models with all lineages combined within a phylogeny [25] we set *Physalis *clades A and C against a background phylogeny of all *Solanum *alleles and the alternative clade (either A or C, depending on which was the test clade). This test also uses the REL framework (see Methods). *Physalis *Clade A had a significantly higher dN/dS ratio (dN/dS Phys Clade A = 2.19; CI = 2.03, 2.36) than all background lineages (*Solanum *plus *Physalis *clade C, dN/dS = 0.70; CI = 0.73, 0.77; Table 4). The branch extending to *Physalis *Clade A had the greatest dN/dS estimate (*Physalis *Clade A Branch dN/dS = 5.18; CI = 1.64, 10.49) but models where this branch was included either as part of the background or as part of Clade A did not provide a statistically worse fit than models in which the dN/dS ratio for this branch was estimated independently (Table 4). Likelihood ratio tests and AIC scores show that models with *Physalis *Clade A specific selection provide a better fit to the data (Models 3, 4 and 5; Table 4) than the model that assumes a single best global estimate of dN/dS. The same procedure was conducted for *Physalis *clade C and also found significantly increased selection relative to background lineages. For clade C the estimated dN/dS ratio (1.33; CI = 1.17, 1.51) is lower than estimated for clade A and the best fit model does not include its subtending branch (results not shown). *Phyalis *clade B was ignored in this and the following analysis because it contains too few sequences to be informative.

**Table 4:** Clade model likelihood ratio tests comparing *Physalis *Clade A (subtree) and its subtending branch to all other S-RNases^a^

Model		
1) Global dN/dS rate (whole tree)		
Shared Parameters	lnL	AIC
dNdS Clade A = dNdS All^a^	-17167.25	34716.51
Global dN/dS = 0.90; CI = (0.87, 0.94)		
		
**2) Separating Branch Versus Two Clades**		
Shared Parameters	lnL	AIC

dNdSdNdS Shared Clades A + All^a ^= 0.90; CI = (0.86, 0.94)	-17166.63	34717.27
Phys dNdS Branch A = 5.18; CI = (1.64, 10.49)		
LRT *p*-value vs the single rate model = 0.266		
		
**3) Clade A + Branch vs **All^a^		
Shared Parameters	lnL	AIC

dNdS Branch A = Clade A = 2.19; CI = (2.03, 2.36)	-17118.27	34620.54*
dNdS Clade S = 0.70; CI = (0.73, 0.77)		
		
LRT *p*-value vs the single rate model < 0.001		
		
**4) Clade A (subtree) vs Branch + **All^a^		
Shared Parameters	lnL	AIC

Phys dNdS Clade A = 2.04; CI = (2.20, 2.37)	-17118.80	34621.60
Phys dNdS Branch A = dNdS; Clade All^a ^= 0.70; CI = (0.73, 0.77)		
LRT *p*-value vs the single rate model < 0.001		
		
**5) Clade A, *Solanum*, and Branch**		
Shared Parameters	lnL	AIC

Phys dNdS Clade A = 2.04; CI = (2.20, 2.37)	-17118.25	34622.51
dNdS Clade All^a ^0.70; CI = (0.73, 0.77)		
Phys dNdS Branch A = 2.96; CI = (0.83, 6.17)		
		
LRT *p*-value vs the single rate model < 0.001		
		

a) Background branches include all *Solanum *alleles and *Physalis *alleles **outside **of sub-Clade A

Alternative models (2-5) were tested using likelihood ratio tests (LRTs) against the null model 1 where dN and dS rates are shared among all branches on the phylogeny. Significance of *p *≤ 0.05 was determined using χ^2 ^with degrees of freedom (DF) equal to the number different parameters between models.

*Model 3 showing a common dN/dS for Clade A AND the subtending branch has the best fit AIC score sharing a distinct dN/dS.

### Do selected sites differ among lineages?

**Figure 6:**
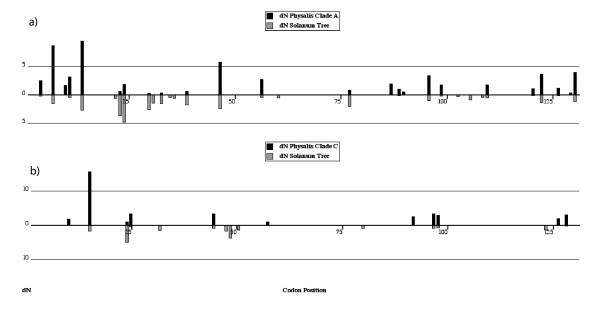
**a) The FEL Clade analysis indicated the 18 codons from *Physalis *Clade A (see Figure 1) that have significantly greater dN/dS values using LRT's and *p*-values ≤ 0.05 and the 10 sites estimated to have greater dN/dS in *Solanum *(sites listed in Table 3)**. b) *Physalis *Clade C shows 9 positively selected *Physalis *sites with only 2 overlapping with Clade A (see also Table 3). Under this model 7 *Solanum *sites show higher dN/dS. The majority of sites favoring greater positive selection in *Solanum *are found in the HVa and HVb regions. Only dN values are reported for Clades A and C (black bars) and *Solanum *(gray bars) as dS values are shared across genera.

It is possible that diversification of different specificities occurs by changes at different sites in different lineages. Using clade-specific FEL (FEL-Clade) based variations of branch models [29,30], we removed the other major *Physalis *clade (A or C from Figure 1) to determine whether each *Physalis *clade exhibits different selected codons relative to the many background lineages from *Solanum*. This test finds 18 codons that have significantly greater dN/dS for Clade A, while 14 show significantly higher selection intensites in *Solanum *than in *Physalis *clade A (Figure 6, Table 3). For *Physalis *Clade C (Figure 6, Table 3), 10 sites show higher dN/dS than in the background lineages from *Solanum *while seven codons are subject to more intense selection in the background lineages than this clade. Sites indicated to be under differential selection in each clade-specific analysis are mostly different (Table 3). The majority of sites found to be under higher levels of positive selection in *Solanum *are in hypervariable regions a and b while sites under greater positive selection in *Physalis *clades A and C are often outside these regions.

### What causes higher dN/dS ratios in *Physalis*?

**Figure 7:**
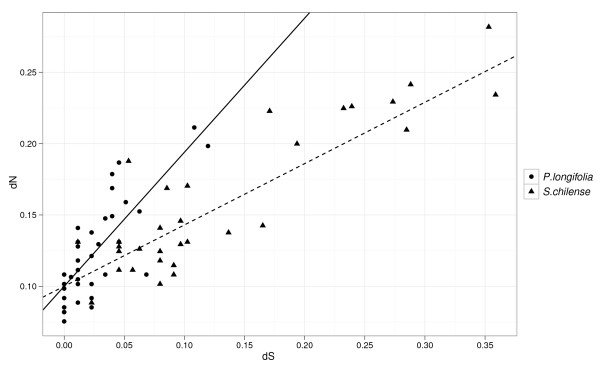
**Linear regression of *P. longifolia *and *S. chilense *terminal branch estimates of dN and dS**. Slopes for *P. longifolia *(solid black line) and *S. carolinense *(dashed line) are 0.77 (S.E. 0.08) and 0.42 (S.E. 0.03) respectively with non-significantly different y-intercepts. Terminal branch estimates of dN and dS were obtained using PAML.

Higher estimated dN/dS ratios in *Physalis *could result from increased fixation of non-synonymous substitutions in *Physalis *because of increased selection, or from fixation of more synonymous changes in the S-alleles of *Solanum *because they are generally older. In order to determine the cause of the difference in estimated selection intensities we used PAML to estimate dN and dS for all terminal branches leading to *P. longifolia *and *S. chilense *alleles, the species which posess the largest S-RNase samples within each genus. Linear regression analysis shows that the Y-intercept (the value of dN when dS = 0) is not different for the two genera (*P. longifolia*: *y*-intercept = 0.097, (S.E. 0.003); *S. chilense*: *y*-intercept = 0.1, (S.E. 0.005)). Apparently, dN = approximately 0.1 is the minimum average divergence among alleles of either genus when synonymous divergence is zero. However, as dS increases, *P. longifolia *alleles show significantly higher accumulation of non-synonymous substitutions as the slopes of the regressions (Figure 7) are significantly different (*P. longifolia = *0.77 (S.E.0.08); *S. chilense *0.42 (S.E. 0.03). For equivalent levels of synonymous divergence, *P. longifolia *alleles have accumulated nearly twice the number of non-synonymous changes as have alleles from *S. chilense*. Quadratic terms are not significant in either genus. Results are nearly identical when all *Physalis *and *Solanum *alleles are used (not shown).

## Discussion

When allele numbers at the S-locus are below equilibrium, as after recovery from a demographic restriction, selection favoring new alleles is expected to increase [2]. We have used a series of statistical methods to determine if the intensity of selection acting on S-RNases differed among taxa and lineages, and whether the number and positions of sites under selection differed. As indicated by the distributions of dN and dS along the entire S-RNase gene in the initial REL models (Table 2), there is a significantly greater dN/dS ratio in *Physalis*. This method is similar to PAML models that begin by categorizing dN and dS rates into discrete distributions, but with the added use of a framework of nested models that compare those rates across two taxa with homologous polymorphism. Subsequent likelihood (PAML) and coalescent (OmegaMap) analyses found more sites under significant positive selection in *Physalis *rendering the second result of the REL analysis somewhat surprising: that no significant difference in the proportion of sites under selection was detected. The REL method may be less sensitive in detecting differences in local processes than in overall selective pressure, but the main difference we can confirm between the genera is in the intensity of selection rather than the proportion of sites subject to it.

We used a novel adaptation of OmegaMap [21] to determine which codons are subject to stronger selection in one genus versus the other. The Markov chain process of the Bayesian method produces a distribution of ω values around a mean for each codon that allows one to establish upper and lower 95% confidence intervals. This feature of Bayesian statistics makes this method useful for hypothesis tests regarding dN/dS ratios across taxa, something that is not possible using existing maximum likelihood methods such as PAML. These tests found 36 codons under significantly higher selection in *Physalis*. We also used an alternative fixed effects maximum likelihood method to compare selective pressures (FEL-CSP) using likelihood ratio tests for increased dN/dS in one genus relative to the other. This method detected roughly half as many sites under differential selection as the Bayesian method, suggesting that either the Bayesian approach is prone to high false positive rates or that the FEL-CSP method has reduced power. Based on our power analysis, we suspect the latter as the Bayesian method appears to perform similarly to a REL method (i.e. PAML). Previous simulations [28] comparing both REL and FEL methods on individual datasets showed that FEL is less powerful when the number of sequences is below 64 as are each of our datasets.

As expected, both the Bayesian and FEL-CSP methods predict that the greatest differences in the magnitudes of positive selection on individual codons occur in the previously identified hyper-variable regions HVa and HVb [31]. The hyper-variable regions are thought to play a major role in determining specificity [31-36]. For example, Matton et al. [35] demonstrated alteration of specificity using mutagenesis experiments involving these hypervariable regions. These studies showed that as few as 4 amino acid changes in corresponding positions of the S_11 _and S_13 _S-RNases of *S. chacoense *could alter specificity to that of the alternative allele. However, entire domain swapping in studies [32,33] using S-RNases of *Petunia inflata *and *Nicotiana alata*, suggest that while HVa and HVb are important, other regions are also likely involved in recognition at least in some alleles or lineages. Consistent with this idea, both codon-based methods used here also show considerable differential selection in the V2 region near the 3' end of the S-RNases, supporting previous analyses of both *Lycium *[16,23] and *Solanum chilense *[12] S-RNases which also found evidence of selection in this region.

The genealogy of S-alleles from *Physalis *suggests that extant S-RNases evolved from only 3 lineages, giving rise to the expectation of strong selection within each of these three clades. Indeed, *Physalis *clade A shows the highest dN/dS as expected during early strong selection on a reduced number of S-alleles. These results suggest that the clade model captures increased post-bottleneck diversifying selection intensities. Clade C also shows increased selection pressure relative to background lineages while clade B contains too few alleles for testing by this method. This test confirms the findings of the REL test but on isolated foreground lineages and shows that selection is generally stronger in each re-diversified clade relative to average selection estimated for background lineages.

All methods used found higher dN/dS ratios in *Physalis*, as expected following a severe reduction in S-allele numbers. However, due to saturation, dN may be more severely underestimated in long branches potentially leading to reduced estimates of dN/dS ratios [19]. Because its alleles are generally older, this could providing a potential alternative to greater selection for lower dN/dS estimates from *Solanum*. We therefore estimated dN and dS at terminal branches for the two species with the most alleles (*P. longifolia *and *S. chilense*) to a) estimate dN and dS in the absence of interspecific branch lengths, b) gain insight into non-synonymous substitution rates of similarly aged S-alleles, and c) estimate recent selection by ignoring internal branches. For alleles separated by equivalent amounts of synonyomous change, *Physalis *alleles have accumulated non-synonymous substitutions at about twice the rate for *Solanum *(Figure 7). Evidence for increased dN/dS ratios is apparent even at relatively low levels of divergence (dN and dS < 0.15). This is strong evidence that saturation of non-synonyous substitutions is not the cause of higher inferred intensity of selection in *Physalis*.

In comparison to tests for increases in selection across the gene or at specific codons, methods for testing whether the same or different codons are under selection in different groups or lineages are considerably less well developed. The FEL-Clade models returned the only evidence suggesting that sites under positive selection in a particular clade might be under neutral or purifying selection in the background phylogeny (Figure 6 and Table 3). FEL-Clade analyses also showed mostly different sites under selection across the two main *Physalis *clades examined (A and C; Table 3). Finding different sites under selection in different clades might indicate that different residues contribute to specificity differentiation in different groups of alleles. However, this finding could also reflect low power to detect selection, given the reduced sample sizes represented within each clade. With low power, the expected overlap in sites predicted to be under selection would also be low [19].

The FEL-Clade models also indicated several sites where the strength of positive selection in *Solanum *was greater than in the contrasted clade (A or C) from *Physalis*. This is in contrast to other methods explored here where all significant differences in the strength of positive selection at specific sites showed increased selection intensity in *Physalis*. If clades differ in sites subject to positive selection, analyses combining all *Physalis *clades might mask these effects while the FEL-Clade method may expose these differences.

## Conclusions

Several methods detected increased selection intensities acting on the alleles from *Physalis *when compared to those from *Solanum*, consistent with recovery from a historical restriction in S-locus diversity in *Physalis*. However, another question, whether the same or different residues were under selection in alleles from the two sources was more difficult to answer. The REL method did not detect a higher proportion of sites under selection in *Physalis *and the method cannot detect whether selection acts on the same or different codons. Other methods found more sites under significant positive selection and higher selection intensities acting on selected sites in *Physalis*, but both may result from increased selection intensities rather than differences in sites subject to positive selection. The FEL clade-specific approach provided some evidence that different sites were under selection in specified *Physalis *clades than across the background *Solanum *alleles but the assumption of this test, that selection on the background clade is uniform, may not hold and these results should be treated cautiously. While the methods explored here for testing differential strengths of selection across a gene or at specific codons appear adequate, further development of statistical methods for testing whether the same or different sites are under selection is needed.

## Methods

### Sequences and Phylogeny Construction

Amino acid and nucleotide S-RNase sequences were obtained from GenBank for 12 *Physalis cinerascens*, 36 *P*. *longifolia*, 17 *Solanum carolinense*, 32 *S. chilense *and one *Antirrhinum hispanicum *(Ahis5) allele used as an outgroup sequence. Automated alignment of the complete dataset containing all S-alleles was performed using ClustalX [37] and manually adjusted using Se-Al v2.0 [38]. A nucleotide alignment was matched with corresponding amino acids to produce a codon alignment using PAL2NAL [39] that resulted in 131 codons. A phylogeny of all S-alleles (*n *= 98) was created using Mr. Bayes v3.1 [40] to generate a 50% majority consensus topology. The analysis was run under a GTR+ Г + I substitution model for 1,000,000 generations, sampling every 100^th ^tree for a total of 10,000 trees. The initial 2501 trees were discarded as the burn-in phase. The remaining trees represent generations on which posterior probabilities were calculated.

Separate datasets were compiled for each genus: one that contained 48 *Physalis *and the other with 49 *Solanum *S-alleles. Corresponding topologies for each dataset were pruned from the Bayesian consensus tree using TreeEdit v1.0a10 [41] to maintain genealogical relationships found when all taxa's alleles were included. The use of 2 species from each genus simply enlarges each dataset as the genealogical patterns exhibited for congeners are shared because of trans-specific polymorphism. The same tree topology for each dataset was used in all subsequent selection analyses that utilize phylogenies unless otherwise stated. A general time reversible (GTR) model of nucleotide substitution is used for all subsequent phylogenetic selection analyses so that direct comparisons can be made across models and datasets. Pairwise nucleotide divergence π was estimated for synonymous and non-synonymous substitutions for all taxa using DNASP 4.0 [42]. Sequence alignments, Newick string tree topologies and HYPHY likelihood functions for *Physalis *and *Solanum *datasets can be found as Nexus files in online Supplementary data.

### Distribution of dN and dS Rates

The most general test of the relative strength of selection across two datasets compares the distribution of synonymous and non-synonymous substitution rates using a random effects likelihood (REL) approach [24] implemented in the program HYPHY [25]. This consists of several nested models for hypothesis testing, similar to the likelihood ratio tests (LRTs) described by Nielsen and Yang [20] and implemented in PAML [43], that begin by estimating general discrete distributions of four rate classes for each dataset. Rate classes are as follows: two bins for negative selection where dS_1 _> dN_1 _and dS_2 _> dN_2_; one for neutral evolution dS_3 _= dN_3 _; and one for positive selection dS_4 _< dN_4_.

Null hypotheses comparing both datasets are as follows: a) H_0_: dN_4p_/dS_4p _= dN_4s _/dS_4s _for the same strength of selection where subscripts indicate bin 4 (dN_4 _> dS_4_) and *Physalis *'p' or *Solanum *'s', b) H_0_: *p*_4p _= *p*_4s _for the same proportion of positively selected sites, c) the same selective regime which combines both a) and b) (H_0_: dN_4p_/dS_4p _= dN_4s _/dS_4s _and *p*_4p _= *p*_4s_), and finally d) H_0_: rates derived from the combined dataset equal to rates estimated for each taxon separately. An independent distribution model of rates that are free to vary for both datasets is set as the alternative hypothesis against which the null model likelihoods (a, b, c and d) are tested. Models are rejected by -2ΔlnL (ΔlnL = the difference in log likelihoods of the two models) where significance is determined by χ^2 ^distribution with the degrees of freedom (df) equal to the difference in the number of parameters between models.

### Codon Selection Estimates

To estimate the ratio (ω) of non-synonymous (d_N_) to synonymous (d_S_) substitutions at individual amino acid sites we first used the program *codeml *in PAML 3.15 [44]. Values of ω < 1 for individual codons indicates purifying selection while sites with ω = 1 are considered neutral. Positive selection at the amino acid level is predicted when ω > 1. A series of nested neutral and selection models first developed by Nielsen and Yang [18] use likelihood ratio tests (LRT) to determine the model that best fits the data. The null model M1 (neutral) constrains all sites to be either of class ω = 0 or ω = 1 while the alternative model M2a (selection) adds a third class in which ω > 1 at individual sites. Model M3 (selection) assumes three discrete site classes (ω_0 _, ω_1_, and ω_2_) with three corresponding proportions (*p*_0_, *p*_1_, *p*_2_) estimated from the data. Models are then compared and rejected by likelihood ratio tests as described in the section above. Sites estimated to be under positive selection are determined by an empirical Bayes approach [44] where posterior probabilities are estimated from rates within each site class. Because we are primarily concerned with comparing posterior probabilities from the robust general discrete (M3) model with a subsequent coalescent analysis, we forgo full analyses including models with more complex rate distributions (i.e. M7 and M8).

The Bayesian coalescent method was conducted using OmegaMap v0.5 [21] which implements a population genetics likelihood approximation to the coalescent to infer recombination and estimate ω. The model of base substitution including transition/transversion rates among codons was adopted from Nielsen and Yang [20]. Rather than using a maximum likelihood approach to estimate the selection parameter, OmegaMap employs a Bayesian method with a Markov Chain Monte Carlo (MCMC) process to estimate posterior distributions of parameters. This allows the use of posterior densities of ω to investigate whether dN/dS is greater at any particular codon in one dataset versus the other without the need for nested models. This can only be done if datasets are the same length, encode for homologous genes, and have reliable alignments of codon positions. By sampling from the distribution of ω values we are able to determine the ratio of ω estimated from *Physalis *relative to *Solanum*. Rejection of the null hypothesis that sites have equivalent ω values is observed when the 95% posterior density of ratios exclude 1 (H_0_: w_1_HPD w_2_HPD = 1).

Rather than estimating ω for each dataset using a variable model along pre-defined blocks of adjacent codons, we assumed an independent model for each site with an improper inverse distribution of rates. The MCMC chain was iterated over 500,000 generations sampling every 100^th ^generation. We ran each dataset twice to check for convergence and removed a burn in of 50,000 generations using R http://www.r-project.org/. The chain generates upper and lower posterior densities (highest posterior density HPD) to determine mean point estimates of ω at each codon position for each dataset. Because the independent model is computationally intensive, we ran the OmegaMap analyses using the Cornell BioHPC server http://cbsuapps.tc.cornell.edu/omegamap.aspx. The upper and lower HPD of ω values from each dataset were then combined and re-sampled after a burn in of 25,000 generations to get HPD's and the geometric mean for the ratio of ω's using R.

### FEL-CSP (Fixed Effects Likelihood-Compare Selective Pressures)

We also used a fixed-effects likelihood (FEL) method to infer differential selection at individual sites among datasets [25]. FEL differs from the REL type models of PAML and the coalescent method of OmegaMap in that dN and dS are estimated at individual sites directly rather than using pre-defined distributions of rates [24]. Alignments of each dataset were first used to estimate global parameters such as nucleotide frequencies, topology, and branch lengths. We use separate trees for each dataset (rather than a single phylogeny including both genera). These parameters were then fixed throughout the selection estimate procedure. The null model H_0_: dN_1_/dS_1 _= dN_2_/dS_2 _and alternative model H_A_: where dS_1_, dN_1_, dS_2_, dN_2 _are free to vary are fitted to every codon and, because they are nested, likelihood ratio tests can be used to determine significantly different selection pressures on individual sites. We estimated selection using the *CompareSelectivePressure *batch file in HYPHY v0.99. Actual dN/dS values for each dataset were then checked for any potential false positive estimates of differential positive selection. Here it is possible for the model to reject the null hypothesis that dN/dS ratios are equivalent across datasets but codons may not actually have ω estimates > 1.

We conducted simulations for *Physalis *and *Solanum *datasets independently to determine the power of the FEL test for given *p*-values. We simulated 100 replicates of each dataset and corresponding phylogeny using the site-by-site rate estimates from the FEL method with 25% of sites evolving neutrally. This produced 13100 sites with non-zero rates (131 codons × 100 replicates) to estimate false positive rates over bins of *p*-values of width 0.01. The power analysis was conducted using a batch command program in the HYPHY v0.99 package.

### Lineage-specific selection pressures

A phylogeny of *Physalis *and *Solanum *compartmentalized into all *Solanum *lineages versus *Physalis *clade A and its subtending branch was used to determine equality of dN/dS between them. *Physalis *clade A represents the largest re-diversification among *Physalis *S-alleles, and this method compares rate estimates for one specified clade against those for a background phylogeny. The HKY85 model of nucleotide substitution was used along with phylogenies containing all *Solanum *S-RNases (49) and the S-RNases found within clade A (Figure 1). Comparison among five models using LRT's are as follows: Model 1) allows one global dN/dS value, Model 2) constrains the specified subclade and background dN/dS values to be equal but adds a new parameter for dN/dS along the branch leading to the clade. Model 3) constrains dN/dS values of the specified clade and its subtending branch to be equal but allows background branches to have a distinct dN/dS value. Model 4) constrains background branch's dN/dS and the subtending branch to be equal while the clade is allowed to vary, and Model 5) allows all compartments (specified clade, its subtending branch, and background branches) to have dN/dS values free to vary. Log likelihood scores were used to determine best fit models and Akaike information criterion (AIC) values were used to adjust for differences in parameters among likelihood ratio tests [25]. The process was then repeated with *Physalis *clade C compared to background lineages from Solanum. *Phyalis *clade B contains too few alleles for useful analysis by this method.

### FEL-Clade Test (subtree selection comparison)

To ask whether different codons were under selection in different lineages we used a FEL approach comparing the selection on individual codons in background lineages with that on a particular *Physalis *clade (A or C). In this case the alternative *Physalis *clade (A or C) was included as part of the background phylogeny. For the class of codons with dN/dS > 1, the null model H_0 _has 3 rate classes for each codon: dN for the background lineages = dN for the *Physalis *clade of interest, dS background lineages = dS *Physalis *clade of interest, dN/dS background lineages = dN/dS *Physalis *clade of interest. The alternative hypothesis H_A_: has one rate class for dN for all background lineages, another dN rate class for *Physalis *clade being compaired, a single dS rate for all lineages, and one dN/dS for all background lineages, and another dN/dS > 1 ratio for the *Physalis *clade of interest. Likelihood ratio tests are conducted for each codon position where significance is determined at the *p *≤ 0.05 level.

## Abbreviations

SI: self-incompatibility; dN: non-synonymous substitution; dS synonymous substitution; REL: random effects likelihood; FEL: fixed effects likelihood; LRT: likelihood ratio test; HPD: highest posterior density; MCMC: Markov Chain Monte Carlo

## Authors' contributions

TP carried out study design, sequence alignment, statistical and genetic analyses, manuscript preparation and editing. JRK assisted in study conception, statistical analysis, and manuscript preparation and editing. All authors read and approved the final manuscript.
